# Identification of strain-specific cues that regulate biofilm formation in *Bacteroides thetaiotaomicron*

**DOI:** 10.1128/spectrum.03419-24

**Published:** 2025-08-18

**Authors:** Robert W.P. Glowacki, Morgan J. Engelhart, Jessica M. Till, Anagha Kadam, Ina Nemet, Naseer Sangwan, Philip P. Ahern

**Affiliations:** 1 Department of Cardiovascular and Metabolic Sciences, Cleveland Clinic Research, Cleveland Clinic 2569, Cleveland, Ohio, USA; 2 Center for Microbiome and Human Health, Cleveland Clinic Research, Cleveland Clinic 2569, Cleveland, Ohio, USA; 3 Department of Molecular Medicine, Cleveland Clinic Lerner College of Medicine, Case Western Reserve University, Cleveland, Ohio, USA; Hong Kong University of Science and Technology, Hong Kong, Hong Kong

**Keywords:** microbiome, bile acids, biofilms, short-chain fatty acids

## Abstract

Members of the gut microbiome encounter a barrage of host- and microbe-derived microbiocidal factors that must be overcome to maintain fitness in the intestine. The long-term stability of many gut microbiome strains within the microbiome suggests the existence of strain-specific strategies that have evolved to foster resilience to such insults. Despite this, little is known about the mechanisms that mediate this resistance. Biofilm formation represents one commonly employed defense strategy against stressors like those found in the intestine. Here, we demonstrate strain-level variation in the capacity of the gut symbiont *Bacteroides thetaiotaomicron* to form biofilms. Despite the potent induction of biofilm formation by bile in most strains, we show that the specific bile acid species driving biofilm formation differs among strains and uncover that a secondary bile acid, lithocholic acid, and its conjugated forms potently induce biofilm formation in a strain-specific manner. Additionally, we found that the short-chain fatty acid, acetic acid, could suppress biofilm formation. Thus, our data define molecular components of bile that can promote biofilm formation in *B. thetaiotaomicron* and reveal that distinct molecular cues trigger the induction or inhibition of this process. Moreover, we uncover strain-level variation in these responses, thus identifying that both shared and strain-specific determinants govern biofilm formation in this species.

**IMPORTANCE:**

In order to thrive within the intestine, it is imperative that gut microbes resist the multitude of insults derived from the host immune system and other microbiome members. As such, they have evolved strategies that ensure their survival within the intestine. We investigated one such strategy, biofilm formation, in *Bacteroides thetaiotaomicron*, a common member of the human microbiome. We uncovered significant variation in natural biofilm formation in the absence of an overt stimulus among different *B. thetaiotaomicron* strains and revealed that different strains adopted a biofilm lifestyle in response to distinct molecular stimuli. Thus, our studies provide novel insights into factors mediating gut symbiont resiliency, revealing strain-specific and shared strategies in these responses. Collectively, our findings underscore the prevalence of strain-level differences that should be factored into our understanding of gut microbiome functions.

## INTRODUCTION

*Bacteroides thetaiotaomicron* is a prominent member of the microbiome present in the human gastrointestinal tract (1). Given its abundance in human populations and genetic tools for its manipulation, the mechanisms through which it competes for nutrients in the intestine and imparts beneficial features to the host have been studied in detail (2–12). Although some mechanisms that foster intestinal resilience in *B. thetaiotaomicron* have been uncovered, there remains a void in our understanding of how *B. thetaiotaomicron* can persist in a dense, competitive environment in the face of ecological challenges such as host-produced anti-microbial peptides (13) and reactive oxygen species (14). One mechanism that provides resistance to a variety of stressors in microorganisms is biofilm formation (15, 16). Biofilms are aggregates of microbial cells embedded in extracellular polymeric substances that can shield the microbes from harmful agents and, as such, represent a lifestyle that allows microbes to survive and thrive in harsh environments (16, 17). Although biofilm formation has not been extensively studied in symbiotic members of the microbiome, recently, it has been uncovered that the type strain of *B. thetaiotaomicron* (*Bt*), *Bt*-VPI-5482, forms robust biofilms in response to purified bile, but not in the absence of an overt stimulus (so-called “natural” or “inherent” biofilm-forming capacity) (18). Seminal studies of this process have uncovered the genetic circuitry that regulates aspects of biofilm formation in *Bt*-VPI-5482, revealing pathways that mediate repression of inherent biofilm-forming capacity (19, 20), or promotion of biofilm formation in response to bile (18, 21), highlighting that biofilm formation appears to be critically tuned to environmental cues. These cues may be important in the context of disease states such as inflammatory bowel disease (IBD), where patients with IBD and related intestinal mucosa-altering conditions were found to have increased biofilms within the intestines (22). Interestingly, fluctuations in the bile acid pool have been associated with differences in disease progression within IBD patients (16, 23), suggesting a potential role for bile and bile acids in promoting biofilm formation.

Despite the clear impact of bile on biofilm formation, several important questions have remained unanswered. Bile is a mixture of many distinct compounds, including retinol (24), bile acids (25), cholesterol (26), bilirubin (27), and more. However, the precise molecular components of bile that promote biofilm formation in *B. thetaiotaomicron* have not been defined. Moreover, as our appreciation of the prevalence and importance of strain-level variation in gut microbes with respect to microbial fitness and beneficial host effects grows (28–31), it is important that such characteristics are determined in multiple strains to determine if they represent broad features of a species or strain-specific pathways that promote strain fitness in a given context. In addition, the existence of inhibitors of biofilm formation in *B. thetaiotaomicron* is unknown, creating a gap in our understanding of the environmental signals that regulate the formation of biofilms. Given that biofilm formation can contribute to microbial pathogenesis, and *B. thetaiotaomicron* also harbors pathogenic potential in the intestine (32–36), understanding how biofilm responses are initiated and repressed, and how this varies depending on the nature of the strain of interest may be important for strategies that aim to modulate the function of the microbiome for therapeutic purposes.

Here, we investigated a panel of human gut-derived *B. thetaiotaomicron* strains to examine their intrinsic biofilm-forming abilities, representing several strains whose biofilm-forming capacity was unknown. We identified a new set of strains with the ability to form robust biofilms in the absence of bile as an inducing molecule, thus uncovering extensive strain-level variation in this response. One of these strains, *B. thetaiotaomicron* VPI-5951 (henceforth referred to as *Bt*-5951) was further investigated. By contrast with *Bt*-VPI-5482, we determined that biofilm formation by *Bt*-5951 was not substantially bile responsive but that lithocholic acid (LCA), a secondary bile acid, and its taurine and glycine-conjugated forms were able to induce *Bt-*5951 to form robust biofilms significantly greater than intrinsic levels, thus revealing variation with respect to the stimuli that induce or expand biofilm formation. Furthermore, we show that the LCA glyco and tauro conjugates, glycolithocholic acid (GLCA) and taurolithocholic acid (TLCA), also act as a biofilm inducer in several strains, suggesting these bile acids represent a biofilm-inducing signal that may act in a similarly broad nature to bile. Moreover, we find that for strain *Bt*-5951, none of the other single bile acid substrates tested is able to support biofilm formation. Additionally, we show that acetic acid, a microbial-derived short-chain fatty acid (SCFA), suppresses the intrinsic biofilm-forming capability of strains *Bt-5951* and *Bt*-0940-1. Thus, our data (i) identify strains of *B. thetaiotaomicron* that had not previously been known to have inherent biofilm-forming capacity, (ii) define key physiologically relevant molecules that govern the formation and inhibition of biofilms in *B. thetaiotaomicron*, and (iii) uncover strain selectivity in the cues that regulate this process, thus providing novel insights into strain-level variation in *B. thetaiotaomicron*. Therefore, our approach expands our understanding of the gut-relevant molecules that coordinate biofilm formation in *B. thetaiotaomicron* and bolsters the utility in studying non-type strains to study the physiology of gut *Bacteroides*.

## RESULTS

### Inherent biofilm formation ability differs among *B. thetaiotaomicron* strains 

**Fig 1:**
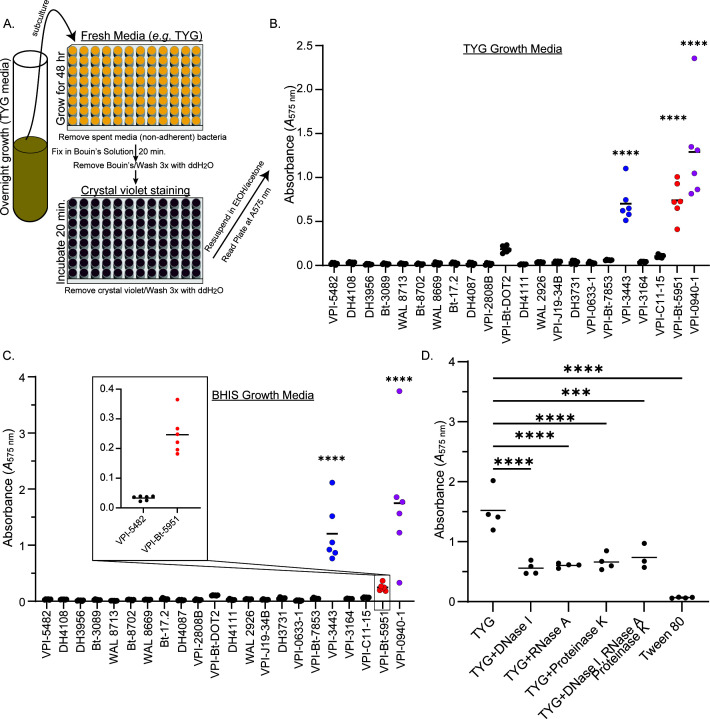
Inherent biofilm formation ability differs among *B. thetaiotaomicron* strains. (**A**) Schematic of the crystal violet-based assay for biofilm formation used throughout this study. (**B and C**) Twenty-two isolates of *B. thetaiotaomicron* were grown in the indicated growth medium, and their biofilm-forming capacity was assessed using a crystal violet-based biofilm assay as described in the schematic in panel **A**. Biofilm formation following growth in (**B**) TYG or (**C**) BHIS was quantified by assessment of absorbance at 575 nm (A575) following crystal violet staining and is shown for each of the indicated strains at 48 hours post-inoculation. The inset in panel **C** shows the magnitude of strain *Bt*-5951 biofilm formation. (**D**) The impact of DNase, RNase, and Proteinase K treatments on biofilm formation in strain *Bt*-5951 was assessed at 48 hours. Data points show individual technical replicates, and bars represent the mean. Graphs are representative of four (**B**) and three (**C and D**) independent experiments, respectively. Bars show the mean, and each point represents an individual technical replicate. Statistical significance was determined using a one-way Analysis of Variance (ANOVA) with Dunnett’s multiple comparisons test, comparisons made to strain *Bt*-VPI-5482 in panels B and C and to TYG in panel D. *P* values of <0.05 were considered statistically significant ***, *P* < 0.001 and ****, *P* < 0.0001.

To identify *B. thetaiotaomicron* isolates with intrinsic biofilm formation capacity, 22 human-derived *B. thetaiotaomicron* isolates were grown on tryptone-yeast extract-glucose (TYG) medium without the presence of known *Bacteroides-*specific biofilm inducers (18, 37, 38) (Fig. 1A). While 17 of the *B. thetaiotaomicron* strains failed to form potent biofilms in TYG media across multiple experiments, three strains, *Bt-*5951, *Bt*-0940-1, and *Bt*-3443, all formed robust biofilms compared to the type strain, VPI-5482 (*Bt*-VPI-5482), while strains VPI-*Bt*-DOT2 and VPI-C11-15 displayed weaker (not statistically significant) biofilm-forming capacity, although above background (Fig. 1B; Fig S1A through C). To determine if this was TYG-media specific or if it represents an intrinsic feature in these strains, we grew these strains in brain-heart infusion-supplemented (BHIS) media and found similar biofilm-forming patterns among strains (Fig. 1C), albeit with differing magnitudes of biofilm formation than that seen in TYG, where some strains maintained a noticeable increase in biofilm formation over strain VPI-5482, although not reaching statistical significance in all cases. We next selected strain *Bt-5951* as a representative strain for further study due to its intrinsic biofilm-forming capabilities and its potential for future follow-up studies due to its genetic tractability, while strain *Bt*-0940-1 and *Bt*-3443 have proved largely recalcitrant to genetic modification to date. Next, these intrinsic biofilms formed by strain *Bt-5951* were treated with DNase, RNase, or Proteinase K, revealing that all three treatments substantially impaired biofilm formation of TYG-grown *Bt-*5951 (Fig. 1D), in keeping with the known role for their targets in the establishment of a biofilm (39). With all three enzymes, residual biofilm was seen, suggesting other factors such as carbohydrates or lipids could be incorporated into these intrinsic biofilms of *Bt-*5951 as shown in other species of bacteria (17, 40). Additionally, supplementation of the media with the nonionic surfactant Tween 80 was able to completely inhibit biofilm formation (Fig. 1D), similar to previous reports of nonionic surfactants inhibiting biofilm or cell attachment, suggesting this is a *bona fide* biofilm structure (41).

### *B. thetaiotaomicron* strain 5951 does not form biofilms in response to bile

**Fig 2:**
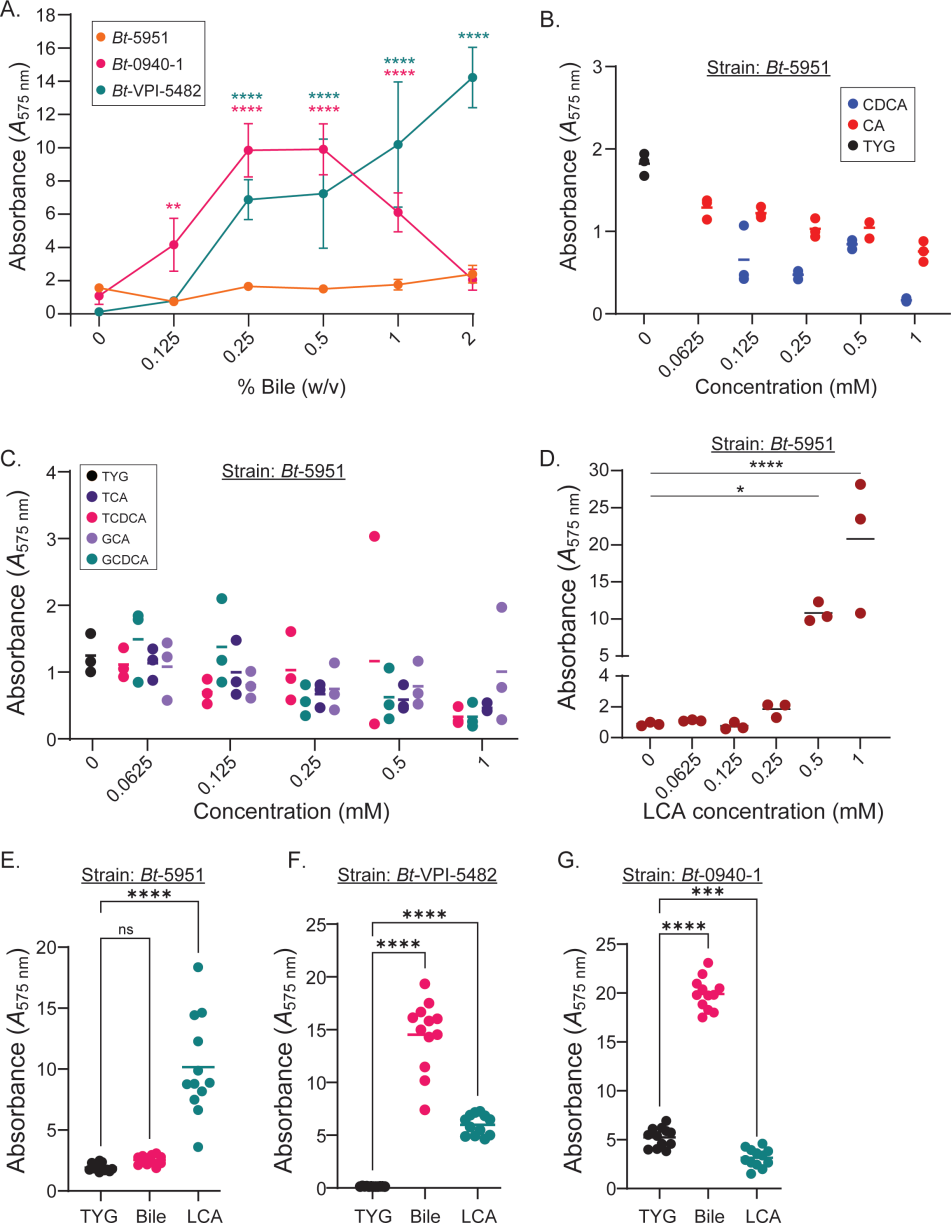
The secondary bile acid lithocholate elicits biofilm formation in strain *Bt*-5951. (**A**) Bile-induced biofilm formation at 48 hours in *Bt* strains 5482, 5951, and 0940-1. (**B**) Biofilm formation dose response at 48 hours for strain *Bt*-5951 grown in TYG media containing primary bile acids cholic acid (CA) or chenodeoxycholic acid (CDCA). (**C**) Biofilm formation dose response at 48 hours for strain *Bt*-5951 grown in TYG media containing taurine and glycine conjugated bile acids taurocholic acid (TCA), taurochenodeoxycholic acid (TCDCA), glycocholic acid (GCA), or glycochenodeoxycholic acid (GCDCA). (**D**) Biofilm formation dose response at 48 hours for strain *Bt*-5951 grown in TYG media containing LCA. (**E–G**) Biofilm formation in TYG media, or TYG media supplemented with 1% wt/vol bile, or 0.5 mM LCA in strains *Bt*-5951 (**E**), strain *Bt*-VPI-5482 (**F**), strain *Bt*-0940-1. (**G**) All biofilms were measured after 48 hours of growth. Biofilm formation was assessed by crystal violet biofilm staining. Graphs are representative of three independent experiments in panels **A and E**, while in panels **B–D**, the dose response is representative of two independent experiments (data for 0.5 mM for all bile acids are independent experiments). Data points show the mean of an average of four technical representatives from at least three separate experiments, error bars show SD (**A**), or bars show the mean, and each point represents an individual technical replicate in panels **B–D**. Statistical analysis in panel **A** was performed using a two-way ANOVA with Dunnett’s multiple comparisons test, comparisons made to the 0% group for a given strain. Statistical analysis in panel **D** was performed using one-way ANOVA with Dunnett’s multiple comparisons test, comparisons made to the 0 mM LCA group (TYG media without LCA supplementation). Data in panel **E** show the mean of 12 technical replicates, and statistical analysis was performed using one-way ANOVA with Dunnett’s multiple comparisons test, comparisons to TYG. *P* values of <0.05 were considered statistically significant, *, *P* < 0.05, ****, *P* < 0.0001. Significance asterisks in panel **A** are color coded to match the strain.

Given the striking capacity of bile to induce biofilm formation in *Bt*-VPI-5482 (18), we sought to address whether biofilm formation capacity in response to bile was a generalizable feature of *B. thetaiotaomicron* species. We initially focused on the following strains: the type strain *Bt*-VPI-5482 as a positive control and two of the intrinsic biofilm formers *Bt-*5951 and *Bt*-0940-1 as we reasoned that inherent biofilm formers may not be dependent on external cues. Strikingly, strain *Bt-*5951 did not increase biofilm production with bile supplementation of up to 2% wt/vol (Fig. 2A) by contrast with the type strain, *Bt*-VPI-5482, and strain *Bt-*0940-1 both of which showed substantial induction in biofilm formation in a dose-dependent manner (Fig. 2A). Notably, unlike strain *Bt*-VPI-5482, bile-induced biofilm formation in strain *Bt-*0940-1 was inhibited at higher concentrations of bile (2% wt/vol; Fig. 2A), likely due to viability issues as higher doses of bile were associated with reduced CFU counts (not shown). Furthermore, the bile-induced *Bt*-VPI-5482 and *Bt*-0940-1 biofilms were treated with DNase, RNase, and Proteinase K, and we observed significant decreases following treatment with DNase and Proteinase K, as previously reported for bile-induced biofilms (Fig. S2A and B), further validating our findings. These data suggest that strain *Bt*-5951 does not form biofilms in response to bile, contrasting with other strains, and/or it requires higher levels of the same stimulus that stimulates biofilm formation in other strains, but insufficient levels are present in bile.

### Lithocholic acid is a potent biofilm inducer in *B. thetaiotaomicron*

As bile is a complex mixture of cholesterol and lipid molecules, bile acids (both host primary and microbiota-generated secondary bile acids), vitamins, bilirubin, and other trace compounds (24–27, 42), we reasoned that strain *Bt*-5951 may respond to a bile acid species that was either not present or under-represented in the bile mixture we used to induce biofilm formation in *Bt*-VPI-5482. To identify components of bile that have the capacity to induce biofilm formation, we further tested the capacity of individual bile acids and their glycine and taurine conjugates (i.e., bile acid salts) that are commonly found in the intestines through the actions of the liver or the gut microbiome (43, 44) to elicit a biofilm response in strain *Bt*-5951. First, we tested two primary, host-derived, bile acids: chenodeoxycholic acid (CDCA) and cholic acid (CA) and observed no change in the amount of biofilm formed when compared to control media (note, the lowest concentration of CDCA tested [0.0625 mM] yielded inconsistent data across replicates and is not shown given its variability; Fig. 2B). Next, we tested glycine and taurine-conjugated forms of CDCA and CA, and again, we did not observe a statistically significant increase in biofilm formation (Fig. 2C). As with *Bt-*5951, strains *Bt*-0940-1 and *Bt*-VPI-5482 showed a similar trend with no significant biofilm induction in response to most of the individual tested bile acids (Fig S2C and D); it should be noted that the response to particular bile acids was somewhat inconsistent for these two strains (data for HDCA and UCDA were not consistent across experiments in *Bt*-VPI-5482 with no effects seen in some experiments and a slight increase in another; data for glycocholic acid [GCA] were not consistent across experiments in *Bt*-0940-1 with no effects seen in some experiments and a slight increase in another).

Finally, we tested secondary bile acids produced by microbial modification of host-derived bile acids and observed that LCA induced a nearly 400% increase in biofilm formation over that seen for an intrinsic biofilm at a concentration of 1 mM for *Bt*-5951 (Fig. 2D). This effect was specific to LCA as other secondary bile acids tested, including deoxycholic (DCA), taurodeoxycholic acid (TDCA), ursodeoxycholic acid (UDCA), and hyodeoxycholic acid (HDCA), did not elicit significant increases in biofilm formation (Fig. S2E), although we cannot exclude that additional microbiome-produced bile species could mediate similar effects or that they are important in other contexts. Due to its low solubility, LCA precipitates from the solution over time at concentrations of 1 mM and higher. However, HDCA and UDCA also form precipitates and do not stimulate biofilm formation (Fig. S2E), demonstrating that precipitation alone is not responsible for the observed biofilm induction. Moreover, at 0.5 mM, we did not observe precipitation of LCA, yet saw large increases in biofilm formation, further reinforcing that LCA itself, rather than the presence of precipitate, is responsible. Furthermore, LCA alone controls without bacteria present also precipitated at 1 mM but had low background compared to when bacteria were present (Fig. S2F), confirming that the precipitated LCA itself did not contribute substantially to the signal. Finally, the solvents used to prepare these secondary bile acid stocks, dimethyl sulfoxide (DMSO) or ethanol (EtOH), did not significantly impact biofilm formation when added to TYG, reflecting that LCA is sufficient to cause biofilm formation irrespective of the specific solvent present (Fig. S2G). Lastly, despite an advertised purity of ≥95%, an independent assessment of LCA purity in-house (not shown) showed that it was composed of ~50% LCA and ~50% CA. However, we found no impact of CA on biofilm formation for our strains tested (Fig. 2B and Fig. S2C and D), allowing us to attribute the induction to LCA.

Given the disparate impact of bile and LCA on biofilm formation in strain *Bt*-5951, we sought to determine the impact of LCA on strains *Bt*-0940-1 and *Bt*-VPI-5482, both of which produce biofilm in response to bile. Thus, strains *Bt*-0940-1 and *Bt*-VPI-5482 were treated with LCA (0.5 mM) or bile as a control. Furthermore, *Bt*-5951 was included as a control for LCA responsiveness. As expected, bile did not induce biofilm formation in strain *Bt*-5951 (Fig. 2E), but robust biofilm formation was seen for strains *Bt*-VPI-5482 (Fig. 2F) and *Bt*-0940-1 (Fig. 2G). LCA induced biofilm formation in *Bt*-5951 (Fig. 2E) and *Bt*-VPI-5482 (Fig. 2F), but it did not increase biofilm formation above that of intrinsic biofilm formation in *Bt-*0940-1 (note, while statistically significant increases in biofilm formation following LCA supplementation were observed in some experiments, the magnitude of biofilm formation—approximately 1.5- to 2.5-fold induction over TYG controls—was markedly smaller than that seen for *Bt*-5951 and *Bt*-VPI-5482 in these experiments (Fig. 2G)).

Due to the strain-selective impact of bile and LCA on biofilm formation in *Bt*-5951, *Bt*-0940-1, and *Bt*-VPI-5482, we sought to expand our analyses in order to determine if these differences were peculiarities of these strains or reflective of the species *B. thetaiotaomicron*. Thus, we treated the panel of *B. thetaiotaomicron* strains with LCA (0.5 mM) or bile (0.5% wt/vol) and assessed biofilm formation. Bile induced a significant increase in biofilm formation for all strains tested (Fig. S3), except strain *Bt*-5951 (Fig. 2E), thus suggesting that bile can be considered a near-universal signal for biofilm formation in *B. thetaiotaomicron*. Additionally, we observed that LCA induced substantial increases in biofilm formation in most tested strains, with the exception of strains *Bt*-0940-1 (Fig. 2G) and *Bt*-3443 (Fig. S3), where LCA elicited a less pronounced increase of variable magnitude and did not consistently cause a significant increase above baseline levels seen in TYG media. Collectively, these data suggest that while both bile and LCA have broad biofilm-inducing capacity in *B. thetaiotaomicron*, strain-level variation with respect to biofilm-inducing cues is evident.

**Fig 3:**
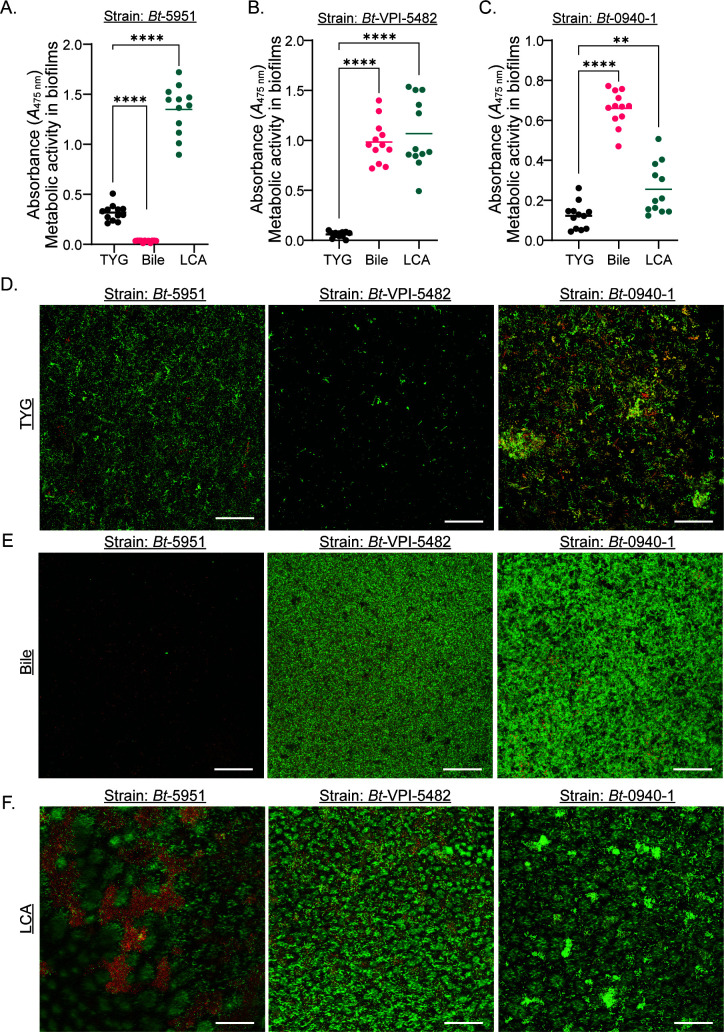
Effects of LCA and bile on biofilm formation in *B. thetaiotaomicron*. (**A–C**) Biofilm formation was assessed using the XTT-based cell metabolic assay following 48 hours of growth in TYG media, or TYG media supplemented with 1% wt/vol bile or 0.5 mM LCA for strains *Bt*-5951 (**A**), *Bt*-VPI-5482 (**B**), and *Bt*-0940-1 (**C**). (**D–F**) Biofilm formation was assessed via confocal imaging of biofilms after 48 hours of growth in TYG media (**D**), or TYG media supplemented with 1% wt/vol bile (**E**) or 0.5 mM LCA (**F**), following staining with SYTO-9 or propidium iodide. Images are processed *Z*-stacks containing both the red (dead or extracellular DNA, propidium iodide) and green (all bacteria, SYTO-9) channels. All images were obtained in the same manner, and the scale bar is equal to 50 µm. Data are representative of four (**A–C**) or one (**D–F**) independent experiments. In panel **A**, bars show the mean and each point represents an individual technical replicate. Statistical significance was determined using one-way ANOVA with Dunnett’s multiple comparisons test, comparisons made to unsupplemented TYG (no bile acid added). *P* values of <0.05 were considered statistically significant, **, *P* < 0.01, ***, *P* < 0.001, and ****, *P* < 0.0001.

The crystal violet assay has limitations as a measure of biofilm-forming capacity (45, 46). Additionally, bile and bile acids like LCA can exhibit growth-limiting or bactericidal activities at higher concentrations due to their detergent-like properties (47, 48). To validate our findings, we assessed bacterial viability in the biofilms using an XTT assay. We focused on strains *Bt*-5951, *Bt*-VPI-5482, and *Bt*-0940-1 as they displayed the inherent, bile-responsive, and/or LCA-responsive biofilm formation phenotypes that were representative of the larger set of tested strains. In strain *Bt*-5951, metabolic activity was observed in TYG media and was increased upon the addition of LCA but not bile supplementation (Fig. 3A), consistent with the observations from the crystal violet assay. For strain VPI-5482, no metabolic activity was evident in TYG alone, but a significant increase was observed in response to both bile and LCA (Fig. 3B). Strain *Bt*-0940-1 showed metabolic activity in TYG alone and in response to bile supplementation and a variable response after LCA supplementation, with increases in some experiments but not in others (Fig. 3C; note, metabolic activity was retained even when not increased following LCA supplementation). Thus, assessment of bacterial viability using the XTT assay was largely consistent with the findings obtained by the crystal violet-based biofilm formation assay. Next, we used confocal laser scanning microscopy (CLSM) to examine the structure of the biofilms. Consistent with other approaches used to examine biofilm formation, only strains *Bt*-0940-1 and *Bt-*5951 had biofilm or biofilm-like structures when grown in TYG (Fig. 3D and Fig. S4A), and only strains *Bt-*0940-1 and *Bt-*VPI-5482 had defined biofilm features when exposed to bile (Fig. 3E and Fig. S4B). Both our crystal violet and XTT assays showed that strain *Bt-*0940-1 formed little or no additional biofilm following stimulation with LCA by comparison to TYG. Our CLSM studies show an increased biofilm compared to TYG, albeit seemingly less than that seen in bile (Fig. 3F and Fig. S4C). In line with our crystal violet and XTT assays, we observed structures consistent with biofilms in strains *Bt*-VPI-5482 and *Bt*-5951 in the presence of LCA (Fig. 3F and Fig. S4C). Based on morphological appearance and viability as assayed in the imaging, it is apparent that bile induces a dense biofilm, while LCA forms a more honeycomb or web-like structure as described in other biofilm structures (49, 50). Thus, these data reinforce the utility of the crystal violet assay as a suitable assessment of biofilm formation in *B. thetaiotaomicron*.

**Fig 4:**
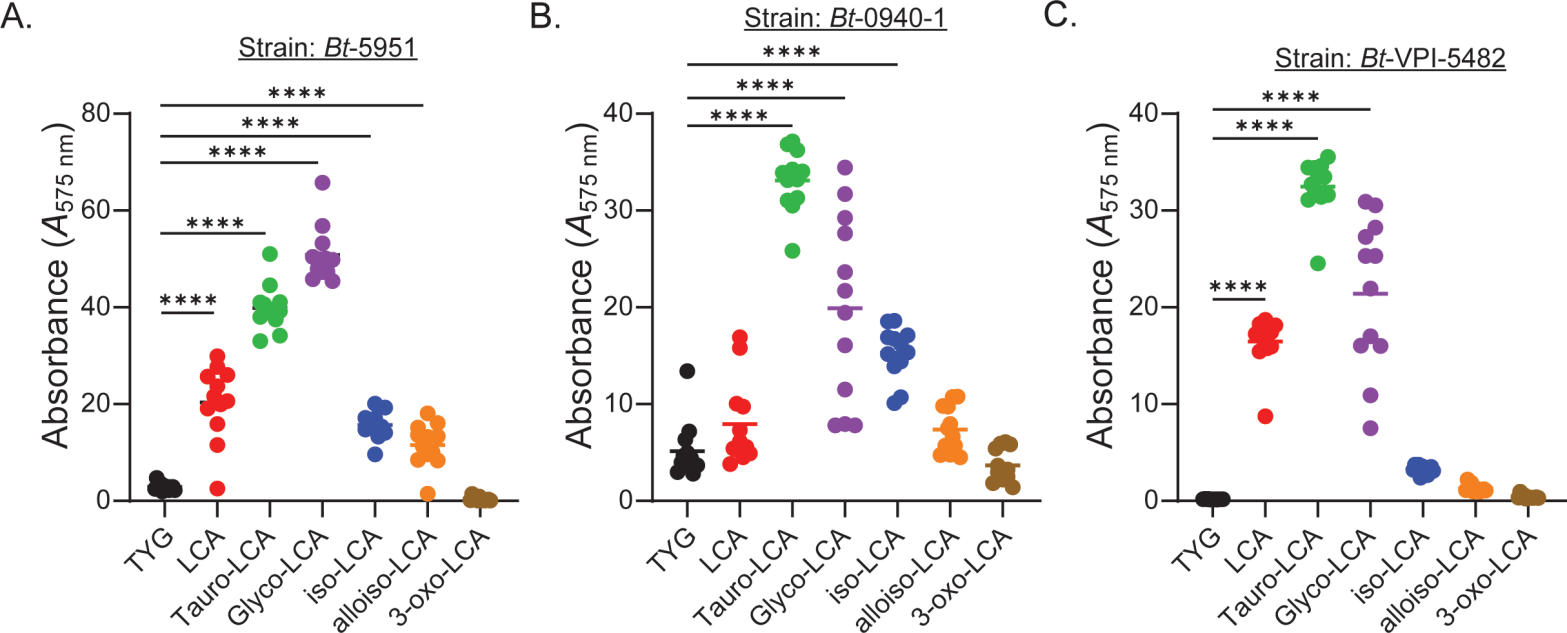
Different forms of LCA mediate biofilm formation in strains of *B. thetaiotaomicron*. (**A–C**) Biofilm formation in the presence of LCA and its epimers or conjugated forms (TYG, LCA, TLCA, GLCA, isolithocholic acid, alloisolithocholic acid, and 3-oxolithocholic acid) for three individual strains of *Bt* was assessed using a crystal violet-based biofilm assay. All bile acids were tested at 0.5 mM, with the exception of isolithocholic acid, which was tested at 0.25 mM due to solubility issues at higher concentrations. Strains shown are (**A**) strain *Bt*-5951, (**B**) strain *Bt*-0940-1, and (**C**) strain *Bt*-VPI-5482. All biofilms were measured after 48 hours of growth. Data are representative of three (**A–C**) independent experiments. Bars show the mean, and each point represents an individual technical replicate. Statistical significance was determined using one-way ANOVA with Dunnett’s multiple comparisons test, comparisons made to unsupplemented TYG (no bile acid added). *P* values of <0.05 were considered statistically significant, ****, *P* < 0.0001.

As LCA exists in a variety of conjugated states *in vivo*, we also tested conjugated forms of LCA, TLCA, and GLCA, as well as its iso-, oxo-, and alloiso-forms for their capacity to promote biofilm formation, again using strains *Bt*-5951, *Bt*-VPI-5482, and *Bt*-0940-1 as their biofilm formation phenotypes were representative of the species as a whole. We observed that both TLCA and GLCA caused a substantial increase in biofilm formation in *Bt-*5951, even greater than those observed with LCA, free acid (Fig. 4A). Additionally, we observed that both iso- and alloiso-LCA produced similar levels of biofilm formation as compared to LCA (Fig. 4A), while 3-oxo-LCA failed to cause an upregulation in biofilm formation over that of TYG (Fig. 4A). These results clearly show that LCA, epimers of LCA, and conjugated forms of LCA promote biofilm formation in *Bt*-5951. As with *Bt-*5951, the conjugated forms of LCA, TLCA, and GLCA induced potent biofilm formation in strains *Bt-*0940-1 and *Bt*-VPI-5482 (Fig. 4B and C), despite the weak/variable response induced by LCA in *Bt-*0940-1. In keeping with *Bt-*5951, iso-LCA induced significant biofilm formation in *Bt-*0940-1 (Fig. 4B) but not *Bt*-VPI-5482 (Fig. 4C). However, neither *Bt-*0940-1 nor *Bt*-VPI-5482 proved responsive to allo-LCA, contrasting with *Bt-*5951 (Fig. 4B and C). Thus, these individual strains respond to both shared and strain-specific cues to coordinate the formation of biofilms.

In an attempt to reconcile the capacity of the identified bile acids to induce biofilm formation in strain *Bt*-5951, with the failure of purified bile to do so in this strain, we performed mass spectrometry-based analysis of the bile used in our assays, as well as our LCA and TYG media (Fig. S5A and B). Although several forms of LCA were detectable in bile at 1% wt/vol, even the combined concentration of all LCA species was at least an order of magnitude lower than the 0.5 mM concentration where we saw effects on biofilm formation (Fig. S5B). This result may reflect why strain *Bt*-5951 did not increase biofilm formation in response to bile but responded robustly to the various forms of LCA tested. Interestingly, of the 51 bile acid species that we attempted to quantify, only 21 were detectable. Of those 21, the only species above 0.5 mM were CA, GCA, TCA, taurochenodeoxycholic acid (TCDCA), GDCA, and TDCA, which lacked potent biofilm induction capacity. Furthermore, our analysis of TYG media revealed that only trace amounts of CA are present in the nanomolar range, and no bile acids that support biofilm formation were detected (not shown).

### Short-chain fatty acids inhibit intrinsic biofilm formation

**Fig 5:**
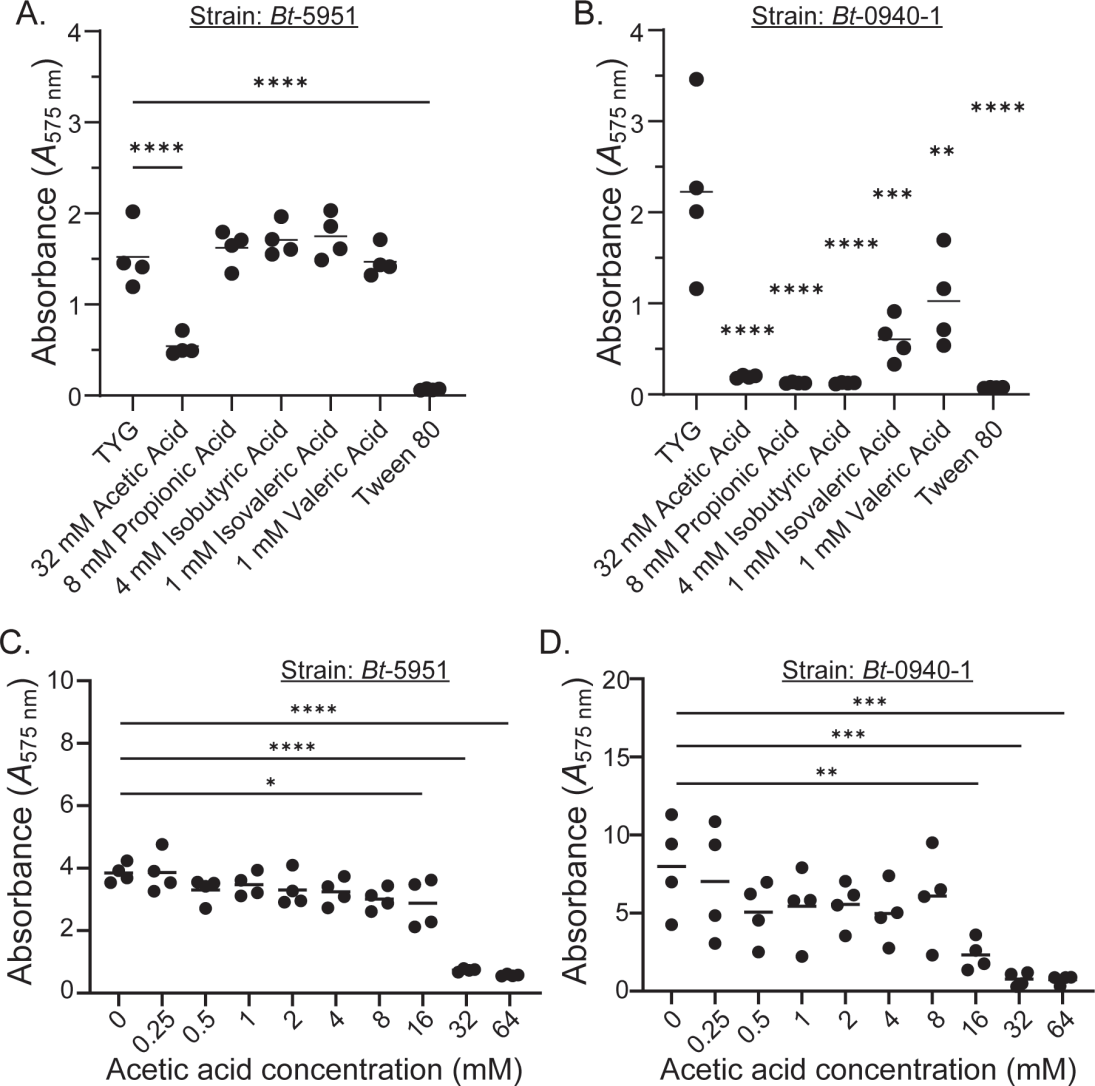
The SCFA acetic acid inhibits the natural biofilm formation of Bt strains. (**A–D**) Biofilm formation was assessed in strains *Bt*-5951 (**A and C**) and *Bt*-0940-1 (**B and D**) using a crystal violet-based biofilm assay in TYG growth media in the presence of the indicated SCFAs (**A and B**), or in a range of doses for the SCFA acetic acid (**C and D**). Data in panels **A and B** are representative of two independent experiments, and data in panels **C and D** are representative of three independent experiments. Tween 80 was added at a concentration of 0.02% vol/vol into TYG media. Bars show the mean, and each point represents an individual technical replicate. Statistical significance was determined in panels **A–D** using one-way ANOVA with Dunnett’s multiple comparisons test, comparisons made to unsupplemented TYG (no SCFA present). *P* values of <0.05 were considered statistically significant, *, *P* < 0.05, **, *P* < 0.01, ***, *P* < 0.001, and ****, *P* < 0.0001.

Due to our observation that bile acids, a prominent microbial-modified compound in the intestines with important physiological implications, had such a profound effect on biofilm formation, we decided to test short-chain fatty acids (SCFAs), another intestinally abundant microbial-derived molecule of physiological relevance (51), for their role in the regulation of inherent biofilm formation within *B. thetaiotaomicron*. We initially tested five SCFAs commonly found in the intestines and tested them at their physiological levels observed within intestinal contents from healthy individuals (51, 52). Importantly, the concentrations tested are not growth inhibitory as we tested them at or below concentrations found in “Gut Microbiota Media,” a medium that supports the growth of intestinal anaerobes, including *Bacteroides* (53), and we did not observe growth defects in our assay. Strain *Bt*-5951 was largely resistant to the effects of most SCFA but was profoundly inhibited by acetic acid (Fig. 5A). By contrast, we observed reductions in the amount of intrinsic biofilm formed for *Bt*-0940-1 in response to most SCFA tested (sensitivity to valeric acid was not evident in all experiments), suggesting that its natural biofilm-forming capacity is exquisitely sensitive to SCFA (Fig. 5B). We next determined the concentration of acetic acid that inhibited biofilm formation by testing the impact of a range of acetic acid concentrations on biofilm formation in strains *Bt*-0940-1 and *Bt*-5951. While both strains showed a significant reduction in biofilm formation at the higher concentrations tested, *Bt*-0940-1 was susceptible to inhibition at lower doses than strain *Bt*-5951 (Fig. 5C and D). SCFAs can act as fuel sources in addition to the modulation of local pH. In order to better understand if this was a pH-dependent phenomenon, we tested the pH of our media with and without acetic acid at these higher concentrations pre- and post-incubation and discovered that at the higher doses of acetic acid, the pH of the media is altered following 48 hours of incubation (Fig. S6A). To determine if altered pH impaired viability, we therefore enumerated CFUs following incubation with our range of acetic acid concentrations and for all of the SCFAs tested. There was no significant reduction in viability for strain *Bt*-5951 following supplementation with acetic acid or other SCFAs (Fig. S6B). Although strain *Bt*-0940-1 did show statistically significant reductions in growth when grown with acetic acid supplementation (Fig. S6C), these differences were likely not the driver of impaired biofilm formation as they were small in magnitude and largely indistinguishable from growth in levels of acetic acid that did not impact biofilm formation, i.e., growth was indistinguishable between 4 mM and 16 mM acetic acid, yet 4 mM acetic acid had little impact on biofilm formation. Thus, while the reduction of pH may act as a trigger for biofilm inhibition, it did not appear to do so via impairing viability.

## DISCUSSION

Members of the gut microbiome face myriad challenges to ensure their fitness in the intestine (54). They must thrive in the face of ever-changing nutrient sources (host diet) (55, 56), competition from other microbes who share their niche (57, 58), antibiotic exposures (59, 60), and a plethora of anti-microbial factors produced by the intestinal immune system (61–63). Despite these challenges, it is evident that many members of this complex intestinal ecosystem have evolved strategies that allow them to overcome these insults. This stability is exemplified by *B. thetaiotaomicron*, a prominent gut symbiont found in a large proportion of human microbiomes (64), that has evolved a variety of strategies that help ensure persistence in the absence of carbon sources of preference (3, 65), as well as modifications to its lipopolysaccharide that provide resistance to anti-microbial peptides (13). *B. thetaiotaomicron* is resistant to the stressors associated with intestinal inflammation (36), but notwithstanding some key insights into the underlying mechanisms involved (13), little is known about how this resilience is mediated. Moreover, the fact that many individuals harbor the same strains of *B. thetaiotaomicron* over extended time periods (66) suggests the existence of strain-specific strategies for survival within the intestine. While the impact of strain-level variation has been extensively studied in microbial pathogenesis (67), such variation in microbiome members has received comparatively little attention, despite evidence of its importance (28, 68–70).

The production of biofilms encased in an extracellular matrix composed of exopolysaccharides, proteins, extracellular DNA, and carbohydrates is a feature of a dense bacterial community that provides microbes a means to resist a variety of factors that could impact their survival. Traditionally, biofilm formation studies have focused on human pathogens due to their capacity to worsen disease outcomes (71–74) and indeed species, such as *Escherichia coli*, *Clostridioides difficile*, *Pseudomonas aeruginosa*, and *Listeria monocytogenes*, experience strain-level variation in their propensity to form biofilms (75–78). While these studies are important, little attention has been devoted to the biofilm-forming abilities of resident human gut microbiome members—despite their intimate relationships within the intestine, where biofilms contact the host mucosal layer but do not interact with the host epithelium during homeostasis (79). Recent studies have uncovered biofilm formation and signals governing biofilm initiation in several human gut commensals, including *Bacteroides fragilis, B. thetaiotaomicron*, *Bifidobacteria*, and *Clostridial* species (18, 37, 38, 80, 81). Biofilm formation may be advantageous to these resident members of the gut microbiome as a means to withstand environmental stressors (15, 16) such as fluctuations in pH and osmolarity (82, 83), alterations to the composition of the bile acid pool (84–86), starvation due to changes in host diet (87, 88), or antimicrobial peptides (89). Here, we have interrogated biofilm formation among different strains of *B. thetaiotaomicron*. Our study has uncovered significant strain-level variation in the propensity to form biofilms in the absence of any currently known inducer molecule or otherwise previously determined factor in separate media conditions, while replicating prior work showing that the type strain of *B. thetaiotaomicron* (*Bt*-VPI-5482) lacks the capacity to form a biofilm in the absence of bile (19). Our studies focused on examining differences in biofilm formation of intrinsic formers (*Bt*-0940-1 and *Bt*-5951) and compared them to the type strain, *Bt*-VPI-5482. While we cannot exclude that the specific media types used masked natural biofilm formation in particular strains, the fact that the same phenomenon was seen using different media types suggests that natural biofilm formation is a feature of select *B. thetaiotaomicron* isolates rather than a widespread feature of the species. Moreover, although no previously described inducing molecule, such as bile, is present in stimulatory amounts in our media, we cannot rule out that a nutrient present in both media types is acting as an unknown trigger of biofilm formation. Thus, the distribution of biofilm-forming capacity may reflect unique strain-specific strategies that have evolved to allow strains to effectively compete for survival in the intestine, while other strains may rely more on the capacity to degrade a wide array of nutrients to maintain fitness (3, 90, 91). Given the potential for *B. thetaiotaomicron* to form abscesses under specific physiological conditions (30, 92), it is possible that this variation in biofilm formation represents a potential virulence feature in these particular contexts. Importantly, the variable distribution of this capacity has implications for studies that aim to infer/predict functions based on phylogenetic relatedness (93). Given the utility in generating accurate predictions of phenotypic outcomes based on the presence/abundance of particular microbes, it is critical to define how such phenotypes vary based on strain identity and how representative a feature of interest is of the species as a whole. Moreover, our data reinforce the need for strain-level resolution in the prediction of community member functions.

Elegant studies have uncovered a role for bile in the induction of biofilm formation among many strains of *B. thetaiotaomicron*, including the type strain *Bt*-VPI-5482 (18–21). To our surprise, despite observing that bile induced or expanded biofilm formation in a large number of strains, including those lacking or possessing intrinsic biofilm-forming capacity, respectively, it had little to no impact on *Bt*-5951. Thus, while bile appears to be a signal for the initiation of biofilm formation among most strains tested, the effect is not uniform, and different strains may respond to distinct molecular cues to initiate this response. Although purified bile had no impact on biofilm formation in *Bt*-5951, the secondary bile acid LCA (a component of bile), its epimers, and its taurine or glycine conjugated forms expanded biofilm-forming capacity, revealing that not only does the capacity to form biofilms vary, but that variation also exists with respect to the molecular signals that initiate the response. Furthermore, LCA promoted a substantial increase in biofilm formation for a large set of strains, including *Bt*-VPI-5482, while again, the effect was not universal, with some strains responding poorly, if at all, to LCA, namely strains *Bt*-0940-1 and *Bt*-3443. Thus, significant strain-level variation exists with respect to the bile acid species to which individual strains are responsive, suggesting that they show distinct biofilm responses depending on the biochemical milieu in which they reside. Additionally, *B. thetaiotaomicron* and related *Bacteroides* are known to have bile salt hydrolases (BSH) that are able to unconjugate secondary bile acids and/or modify these into tertiary bile acids (94–96). Therefore, although our results clearly show that TLCA and GLCA are able to cause a profound increase in biofilm formation, we cannot exclude that the actions of these BSH enzymes are modifying GLCA and TLCA into other forms. These results are interesting in the context of intestinal diseases, as bile acids are known to have large fluctuations in patients with IBD, predominantly in patients with active disease, including diarrhea, where the pool of secondary bile acids is largely diminished while the pool of primary bile acids increases (97, 98). Without these secondary bile acids, it is possible that members of the gut microbiota, such as *Bacteroides*, are lacking important molecular cues that may dictate survival patterns within the intestine. Additionally, recent papers suggest that the repertoire of secondary bile acids and in-turn conjugated secondary bile salts has been understated due to the observation that BSH from the gut microbiome can produce amidated-bile acids through conjugating additional amino acids beyond taurine and glycine (96). These *bsh* gene functions are also important in shaping the gut microbiome and restricting the growth of pathogens like *Vibrio cholerae* (99) and *C. difficile* (85), including in a lithocholic acid-dependent mechanism (100). Beyond bile acids acting as one gut-derived molecule affecting biofilm formation, there exists a plethora of additional molecules and situational dependency whereby substances such as short-chain fatty acids (101, 102) and carbohydrates affect biofilm formation of gut microbiome members (103, 104).

Our data define a role for intestinal bile acids and SCFAs in the regulation of the biofilm-forming propensity of *B. thetaiotaomicron* strains *in vitro* and suggest that these two classes of molecules may operate as signaling molecules that govern biofilm formation in a strain-specific manner. Interestingly, in testing individual bile acids, LCA and its related forms were consistently the only bile acid substrate capable of eliciting or enhancing biofilm formation of the bile acid species we tested. We observed that non-LCA bile acids suppressed or had little impact on biofilm formation for intrinsic biofilm-forming strains, *Bt-*0940-1 and *Bt*-5951. Furthermore, for *Bt*-VPI-5482, no individual bile acid besides LCA and its related forms was capable of stimulating biofilm formation. Notably, bile is known to stimulate biofilm formation in these two strains, and our data show that LCA concentrations are far below those supporting biofilm formation, which suggests that (i) bile-induced biofilms may require more than one bile acid or a net sum of inducing bile acids; or (ii) the non-bile acid fraction of bile, such as retinoic acid (24) or bilirubin (27), may promote a pro-biofilm state. Importantly, in our analysis of bile, no individual LCA compound or the sum of LCA compounds was at levels seen to stimulate biofilm formation. Lastly, in terms of substrates that play a role in the lifecycle of biofilm formation, our work demonstrates that the short-chain fatty acid acetic acid is able to abolish the intrinsic biofilm-forming propensity of strains *Bt-*0940-1 and *Bt*-5951 at concentrations that are physiologically relevant. SCFAs have previously been associated with inhibiting biofilm formation for pathogens (102, 105, 106); however, little is known about their role in biofilms of gut commensals. Given that *Bacteroides* are producers of SCFAs through the breakdown of carbohydrates which primarily leads to the production of acetate (4), it stands to reason that strains may have adapted a positive feedback mechanism whereby they sense acetate levels as a proxy for cell behavior based on nutrient availability in a similar manner as having been seen for bile acid concentrations affecting the gene regulation of carbohydrate metabolism (94). Importantly, all of our data are performed in the context of *in vitro* growth conditions in the background of rich media (TYG); in the context of the gut environment, where nutrients are limited and SCFAs and bile acids are both present, there exists significant future work to delineate the impact of these molecules on biofilm formation *in vivo*. Nevertheless, the ability to sense differences in both the bile acid pool and the SCFA pool during disease states—such as during active IBD—may act as a molecular switch for *B. thetaiotaomicron*, signaling when it should exist in a biofilm for survival versus as a planktonic cell when conditions are favorable.

Collectively, our work provides novel insights into the mechanisms through which *B. thetaiotaomicron* biofilm formation is mediated and uncovers strain-level variation not only in the capacity to form biofilms but also with respect to the signals that coordinate this process. Given that we observed differences at the strain level, our approach underscores the importance of examining physiological responses across strains to gain a better understanding of the complex interplay of the inhabitants of the human gut and not being solely reliant on species-level inferences, as is common in DNA sequencing-based approaches to study the microbiome. These strain-level variations may represent a distinct strategy through which individual strains promote their own fitness in the gut or be reflective of their lifestyle in the intestine and require further investigation.

## MATERIALS AND METHODS

### Bacterial strains and growth conditions

All bacterial strains used in this study are listed in Table S1. *B. thetaiotaomicron* strains were routinely grown in TYG broth in an anaerobic chamber (Coy Manufacturing, Grass Lake, MI) at 37°C in an atmosphere of 5% CO_2_, 5% H_2_, and balance N_2_. TYG was prepared as follows: tryptone peptone (10 g/L; Gibco, Cat. # 211921), bacto yeast extract (5 g/L; BD, Cat. # 212750), glucose (2 g/L; Sigma, Cat. # G8270), cysteine HCl (0.5 g/L; Sigma, Cat. # C1276), 1 M potassium phosphate buffer (pH 7.2, 100 mL/L) made using 1M potassium phosphate monobasic (Sigma, Cat. # P0662), and 1 M potassium phosphate dibasic (Sigma, Cat. # P3786), vitamin K3 (menadione; 1 mL/L of a 1 mg/mL solution; Sigma, Cat. # M5625), TYG salts (40 mL/L) made using MgSO_4_.7H_2_O (0.5 g/L; Sigma, Cat. # M63-500), NaHCO_3_ (10 g/L; Sigma, Cat. # S4019), NaCl (2 g/L; Sigma, Cat. # S3014), FeSO_4_ (1 mL/L of a 0.4 mg/mL stock; Sigma, Cat. # F8048), CaCl_2_ (1 mL/L of an 8 mg/mL stock; Sigma, Cat. # C7902), hemin (0.5 mL/L of a 10 mg/mL solution; Sigma, Cat. # 51280), and vitamin B12 (0.5 mL/L of a 0.01 mg/mL solution; Sigma, Cat. # V2876). For biofilm assays, bacteria were grown in either BHIS broth with hemin and L-cysteine (107) or TYG broth, where indicated. All liquid broth media were filter sterilized through a 0.22 µm filter (Millipore). Media formulations and supplier information for the above-mentioned media can be found in Table S1. For maintenance of *B. thetaiotaomicron* on solid media, brain-heart infusion (BHI) plates supplemented with 10% vol/vol defibrinated horse blood (QuadFive, MT; Cat. # 210-500 mL) were used (“BHI-blood plates” hereafter).

### CFU assay

Enumeration of CFUs was performed by spot-dilution plating on BHI-blood plates following biofilm assays after 48 hours of growth in 96-well plates. Briefly, 10 µL of culture was serially diluted, and then 10 µL of these dilutions were spot plated onto plates in technical duplicate and allowed to incubate for 48 hours anaerobically, followed by visual counting of colonies.

### 96-well biofilm assays

*B. thetaiotaomicron* strains were grown overnight in TYG broth and subcultured into either fresh TYG or BHIS with a wash step in fresh media via centrifugation at 8,000 × g for 3 minutes and resuspended in the appropriate media at an OD_600_ of 0.01. Bacteria were transferred to a 96-well, non-treated polystyrene plate with round wells and flat bottom (Corning, Cat. # CLS3370), and grown in 200 µL of media for 48 hours as indicated without disturbing the plate. Outside-facing wells of the plate were filled with water, and no bacteria were seeded in these wells due to evaporation concerns. Assays involving enzyme treatments of DNase I (Sigma Cat. # 10104159001), RNase A (Thermo Scientific Cat. # J61996) (108), and Proteinase K (Fisher Scientific Cat. # BP1700) (80) were done by adding these enzymes at the time of bacterial seeding into the 96-well plates and were left active for the duration of the 48 hour assay at the following concentration DNase I, 100 U/mL; RNase A, 1 U/mL; Proteinase K, 1 mg/mL. Biofilm staining was performed as described previously (19). Briefly, bacterial supernatant was carefully removed from wells via pipette aspiration, followed by immediate fixing through the addition of 150 µL Bouin’s solution (Sigma, Cat. # HT10132). Fixation was allowed to proceed for 20 minutes, following which the fixative was aspirated, and the wells were subjected to gentle washing with 200 µL ddH_2_O, with a total of three washes performed. A total of 150 µL of a 1% wt/vol Crystal Violet solution (Sigma, Cat. # V5265) was added and stained for 20 minutes, followed by three 200 µL water washes. Stained biofilms were dissolved in a 4:1 ethanol:acetone mixture and were mixed via pipetting until all solids had dissolved. Absorbance was read at 575 nm on a microplate reader (Synergy HT, Biotek/Agilent Systems) using Gen 5 software. For those wells whose *A*_575_ exceeded the linear range of the plate reader, a dilution of the well contents was prepared for *A*_575_ measurement, and the true *A*_575_ was calculated by correcting for the dilution factor.

Measurements of cell viability within the biofilm were performed by using an XTT (2,3-bis-[2-methoxy-4-nitro-5-sulfophenyl]−2H-tetrazolium-5-carboxanilide)-based assay, which facilitates colorimetric detection of the reduction of the tetrazolium salt XTT to formazan by live bacteria. As described above, bacteria were grown in 96-well microplates for 48 hours, and then supernatant was removed and bacteria were washed 2–3 times with phosphate-buffered saline (PBS). PBS (200 µL) containing XTT (0.2 mg/mL; Sigma, Cat. # X4626) and menadione (0.025 mM; Sigma, Cat. # M5625) was added to wells and incubated for 3 hours anaerobically in the dark (109). The absorbances at 475 nm and 650 nm were measured. The total absorbance at *A*_475_ as a measure of cell viability was calculated by subtracting the background absorbance *A*_650_ and the blank (no bacteria added) control wells *A*_475_ values from the observed *A*_475_ measurements of wells with biofilms.

### Testing the impact of bile acids and short-chain fatty acids on biofilm formation

The impact of bile acids on biofilm formation in TYG media was assayed via the addition of the following compounds: sodium cholate hydrate (Sigma, Cat. # C9282) or conjugated bile acids were dissolved in ddH_2_O and diluted in TYG at the indicated molarity in each assay, and the following conjugated bile acids were used: glycocholic acid hydrate (Sigma, Cat. # G2878), sodium glycochenodeoxycholate (Sigma Cat. # G0759), sodium glycocholate hydrate (Sigma, Cat. # G7132), taurocholic acid sodium salt hydrate (Sigma, Cat. # T4009), sodium taurochenodeoxycholate (Sigma, Cat. # T6260), and sodium taurodeoxycholate hydrate (Sigma, Cat. # T0875). For bile acids not readily soluble in water, 100% EtOH or 100% DMSO was used to make stock solutions at high molar concentrations including lithocholic acid (Sigma Cat. # L6250), glycolithocholic acid (Cayman Chemical Cat. # 20273), taurolithocholic acid (Cayman Chemical Cat. # 17275), alloisolithocholic acid (Cayman Chemical Cat. # 29542), isolithocholic acid (Cayman Chemical Cat. # 29545), dehydrolithocholic acid (also known as 3-oxo-LCA; Cayman Chemical Cat. # 29544), deoxycholic acid (Sigma Cat. # D2510), hyodeoxycholic acid (Sigma, Cat. # H3838), ursodeoxycholic acid (Sigma, Cat. # U5127), and chenodeoxycholic acid (Sigma, Cat. # C9377). These solutions were then diluted in TYG to yield working solutions containing 2% vol/vol ethanol or 1% vol/vol DMSO at the highest concentration of these compounds. The compounds were then mixed 1:1 in the well with 2× concentration of TYG with bacteria, yielding a final concentration of 1% or 0.5% vol/vol ethanol or DMSO. To control for ethanol or DMSO affecting biofilm formation, TYG media containing 2% vol/vol ethanol or 1% vol/vol DMSO without added bile salts were used as controls, and comparisons for individual bile acids were made to the appropriate controls. Dilutions were made fresh in all cases and were diluted in the well, as the addition of the stock into TYG caused some of the compound to precipitate. To control for background absorbance caused by this precipitation, blank wells without bacteria were run as controls and stained as above. Biofilm assays were grown for 48 hours prior to staining. As a control for biofilm formation, bile (Sigma Cat. # B8381) was used as described previously for *B. thetaiotaomicron* strains (18). Briefly, bile was added as a percent wt/vol into liquid TYG media at 0.0625%, 0.125%, 0.25%, 0.5%, 1%, or 2% wt/vol to test the response of several isolates of *B. thetaiotaomicron*. For all bulk bile, bacteria were grown for 48 hours prior to assessment of biofilm formation. For SCFA biofilm assays, the compounds were added to TYG as above for bile acids, followed by the addition of bacteria and allowed to incubate undisturbed for 48 hours followed by staining with crystal violet as above. Acids were commercially purchased from the following vendors and used at the indicated concentrations within each assay; acetic acid (Sigma, Cat. # A6283), propionic acid (Sigma, Cat. # 402907), isobutyric acid (Sigma, Cat. # 58360), isovaleric acid (Sigma, Cat. # 129542), and valeric acid (Sigma Cat. # 240370).

### Measurements of pH

The impact on pH of the media in 96-well plates as a result of incubation with the short-chain fatty acid acetic acid (Sigma, Cat. # A6283) was performed using a Reflectometer RQflex 20 instrument (Sigma, Cat. # 1.17246) and pH strips suited for this instrument in the pH range of 4.0–9.0 (Sigma, Cat. # 1.16996). All measurements were done according to the manufacturer’s instructions, and the instrument was calibrated prior to each use using the manufacturer-provided calibration kit. Briefly, the pH of the media was monitored using these strips prior to the addition of bacteria in TYG media only or containing 16, 32, or 64 mM acetic acid and measured again following the 48-hour incubation period.

### Confocal laser scanning microscopy of biofilms

Bacteria were grown anaerobically for 48 hours in TYG media or TYG media plus 1% wt/vol bile or 0.5 mM LCA in 29 mm glass bottom dishes with a 10 mm bottom well with #1.5 glass (Cellvis, Cat. # D29-10-1.5-N). Supernatant was carefully removed, and biofilms were washed twice with PBS. As described previously (110), we added 500 µL of Filmtracer LIVE/DEAD Biofilm Viability strain (Invitrogen, Cat. # L10316) to the dish after being prepared as follows: 3 µL propidium iodide and 3 µL SYTO 9 added per 1 mL of PBS. Dishes were incubated for 30 minutes at room temperature in the dark. The stain was removed, and the dishes were washed three times with PBS. A total of 500 µL of fixative (Biolegend, Cat. # 420801) was added to dishes and incubated for 1 hour at room temperature in the dark. The fixative was removed, and the dishes were washed three times with PBS. One milliliter of PBS was added to the dish, and biofilms were imaged as follows: *Z*-stacks through the entire thickness of the biofilms were acquired using a 40×/NA 1.25 oil objective on a Leica TCS-SP8-AOBS inverted confocal microscope (Leica Microsystems, GmbH, Wetzlar, Germany) utilizing Leica Application Suite X software (LAS X v3.5.7). Biofilms for each condition were grown in triplicate, and four brightest fields from each sample were imaged using the same laser and HyD detector settings across all samples, minimizing signal saturation for both fluorescent probes. Maximum projection images for all the *Z*-stacks were generated using the LASX software. Scalebars in the images are 50 µm wide.

### Bile acid quantification

Bile acids were quantified by a previously published stable-isotope-dilution liquid chromatography mass spectrometry (LC-MS/MS) method (111) with some modifications. Briefly, the internal standard (IS) mix was prepared by mixing D_4_-CDCA, D_4_-CA, D_4_-deoxycholic acid (D_4_-DCA), D_4_-LCA, D_4_-glycochenodeoxycholic acid (D_4_-GCDCA), D_4_-GCA, D_4_-glycodeoxycholic acid (D_4_-GDCA), D_4_-GLCA, D_4_-glycoursodeoxycholic acid (D_4_-GUDCA), D_4_-TCA, D_4_-TDCA, and D_4_-TCDCA. IS mix (80 µL) was added to each sample (20 µL of TYG media and TYG media plus bile at 1% wt/vol). Samples were vortexed and centrifuged (14,000 *× g*, 20 min, 4°C). To cover different concentration ranges of bile acids in the samples, samples were run as undiluted as well as diluted 10× and 100×.

Calibration curves were prepared by mixing known amounts of individual bile acids (BAs; pure chemicals), at seven different concentrations in methanol, and quality control samples were prepared from pooled samples. Both calibration curve standards and quality control samples were processed identically as samples. Calibration curves were built by fitting each analyte concentration to the analyte/IS peak area ratios. LC-MS/MS analysis was performed on the same chromatographic system as described above. An ACE Excel C18-Amide column (75 mm × 2.1 mm; 1.7 µm; Cat. # EXL-1712-7502U, Avantor, Radnor Township, PA) was used for chromatographic separation. A gradient of solvent A (0.1% acetic acid in water) and B (0.1% acetic acid in acetonitrile: methanol 50:50; vol/vol) was used for chromatographic separation with a flow rate of 0.4 mL/min and a 3 µL injection volume. Electrospray ionization in negative ion mode was used with the following multiple reaction monitoring conditions: *m/z* 373.3→373.3 for cholenic acid and 3-keto-cholanic acid; *m/z* 375.3→375.0 for iso-LCA, LCA, allo-iso-LCA, and allo-LCA; *m/z* 377.3→377.3 nor-DCA; *m/z* 387.3→387.3 for 3,7-diketocholanic acid, 3,6-diketocholanic acid, and 12-keto-9,5-cholenic acid; *m/z* 389.3→389.3 for 3-keto-DCA, 6-keto-LCA, 7-keto-LCA, 12-keto-LCA, apo-CA, and 3-keto-CDCA; *m/z* 391.3→391.3 for DCA, HDCA, CDCA, UDCA, iso-UDCA, 7-iso-DCA, iso-DCA, and muro-DCA; *m/z* 401.2→331.1 for triketocholanic acid; *m/z* 403.3→403.3 for 7,12-diketo-LCA, 7-keto-DCA, and takeda ketol; *m/z* 407.2→407.2 for CA, α-muricholic acid (α-MCA), β-MCA, ω-MCA, hyocholic acid, ursocholic acid, and allo-CA; *m/z* 432.3→73.9 for GLCA; *m/z* 448.3→73.9 for GCDCA, GHDCA, GUDCA, and GDCA; *m/z* 455.2→96.9 for LCA-SO4; *m/z* 458.2→73.9 for G-dehydroCA; *m/z* 464.3→73.9 for GCA and GHCA; *m/z* 482.2→123.9 for TLCA; *m/z* 487.2→96.9 for CA-7-SO4; *m/z* 498.2→123.9 for THDCA, TCDCA, TDCA, and TUDCA; *m/z* 514.2→123.9 for TCA, THCA, T-α-MCA, T-β-MCA, and T-ω-MCA; *m/z* 379.3→379.3 for D_4_-LCA; *m/z* 395.3→395.3 for D_4_-CDCA and D_4_-DCA; *m/z* 411.2→411.2 for D_4_-CA; *m/z* 436.2→73.9 for D_4_-GLCA; *m/z* 452.3→73.9 for D_4_-GCDCA, D_4_-GDCA, and D_4_-GUDCA; *m/z* 468.2→73.9 for D_4_-GCA; *m/z* 502.2→127.9 for D_4_-TDCA and D_4_-TCDCA; *m/z* 518.2→127.9 for D_4_-TCA.

### Statistical analysis

Statistical analyses were performed using built-in analysis within GraphPad Prism version 10.2.1. The specific statistical tests used are indicated within the figure legends.

## SUPPLEMENTAL MATERIAL

Fig. S1 spectrum.03419-24-s0001.tif Replicates of biofilm formation in TYG media.

Fig. S2 spectrum.03419-24-s0002.tif Impact of bile on biofilm formation.

Fig. S3 spectrum.03419-24-s0003.tif Bile- and lithocholic acid-induced biofilm formation across all strains.

Fig. S4 spectrum.03419-24-s0004.tif Imaging of bile and lithocholic acid induced biofilm formation.

Fig. S5 spectrum.03419-24-s0005.tif Compositional analysis of bile.

Fig. S6 spectrum.03419-24-s0006.tif Short-chain fatty acids do not substantially affect viability.

Legends spectrum.03419-24-s0007.pdf Supplemental figure legends.

Table S1 spectrum.03419-24-s0008.xlsx Bacterial strains and growth media.

ASM does not own the copyrights to Supplemental Material that may be linked to, or accessed through, an article. The authors have granted ASM a non-exclusive, world-wide license to publish the Supplemental Material files. Please contact the corresponding author directly for reuse.
