# Supplementary figures and images for: Identification of strain-specific cues that regulate biofilm formation in Bacteroides thetaiotaomicron
Source: Microbiol Spectr. 2025 Aug 18;13(10):e03419-24. doi: 10.1128/spectrum.03419-24 (PMC12502800; doi:10.1128/spectrum.03419-24)

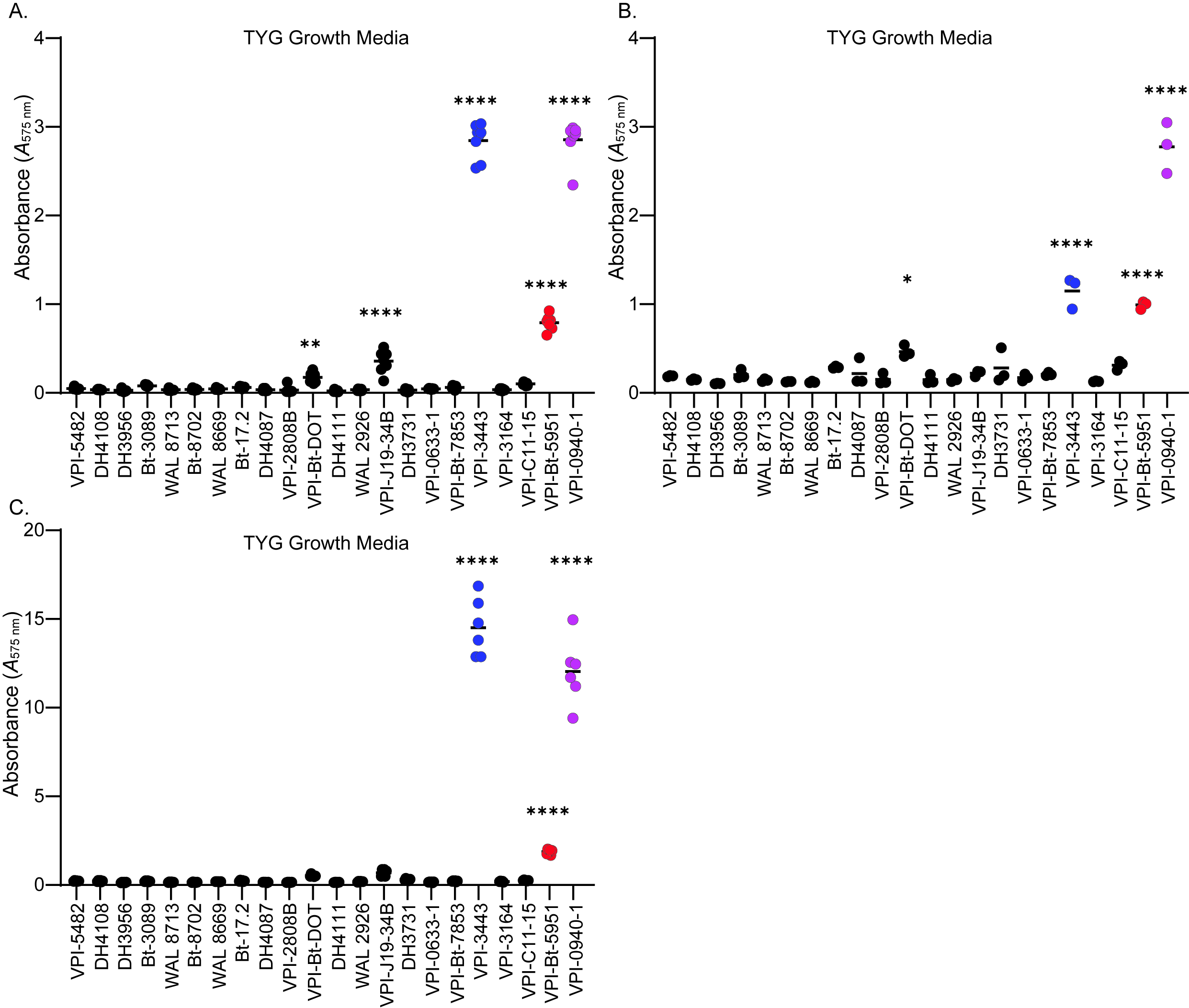

Supplement: Fig. S1 — Replicates of biofilm formation in TYG media. [file spectrum.03419-24-s0001.tif]

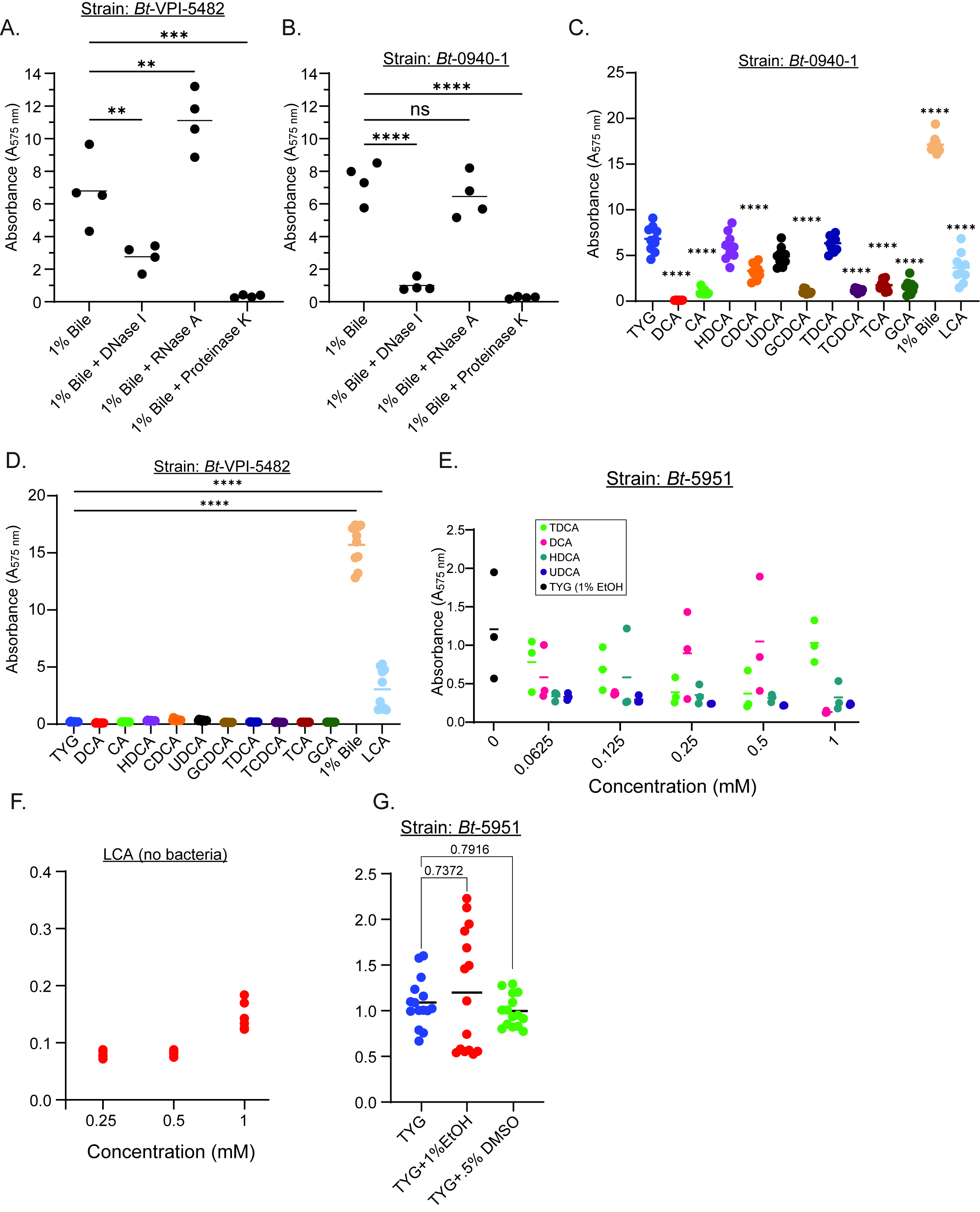

Supplement: Fig. S2 — Impact of bile on biofilm formation. [file spectrum.03419-24-s0002.tif]

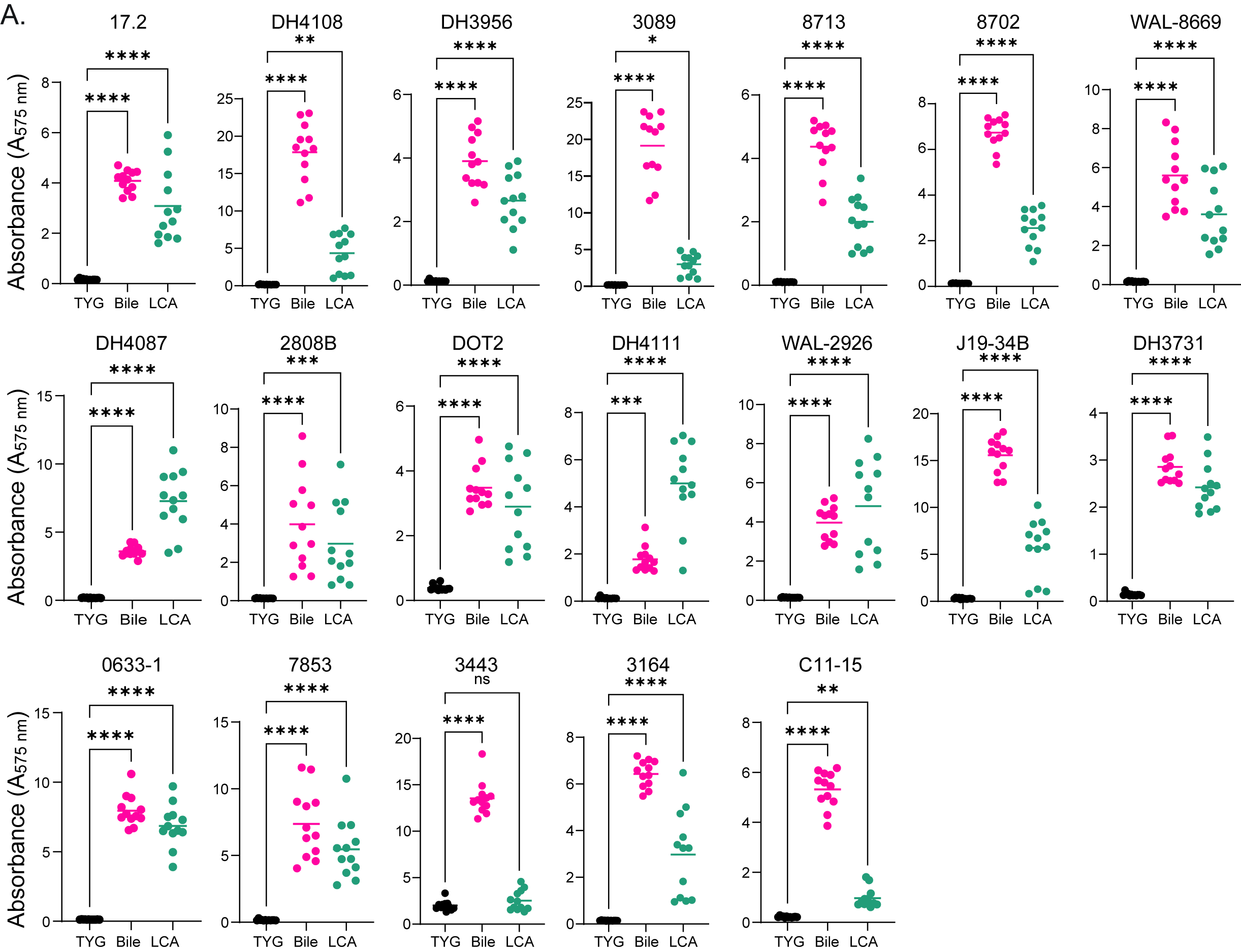

Supplement: Fig. S3 — Bile- and lithocholic acid-induced biofilm formation across all strains. [file spectrum.03419-24-s0003.tif]

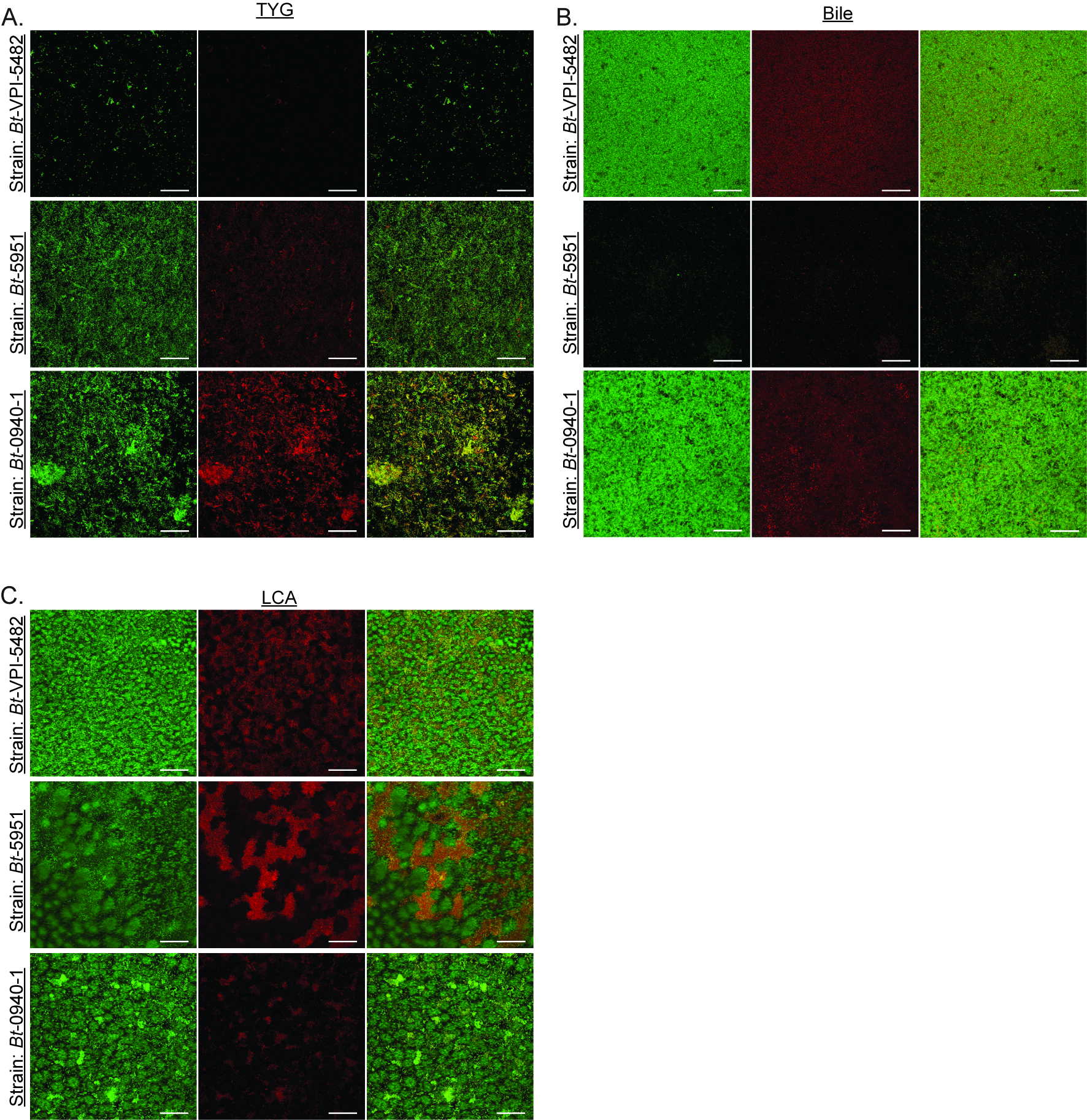

Supplement: Fig. S4 — Imaging of bile and lithocholic acid induced biofilm formation. [file spectrum.03419-24-s0004.tif]

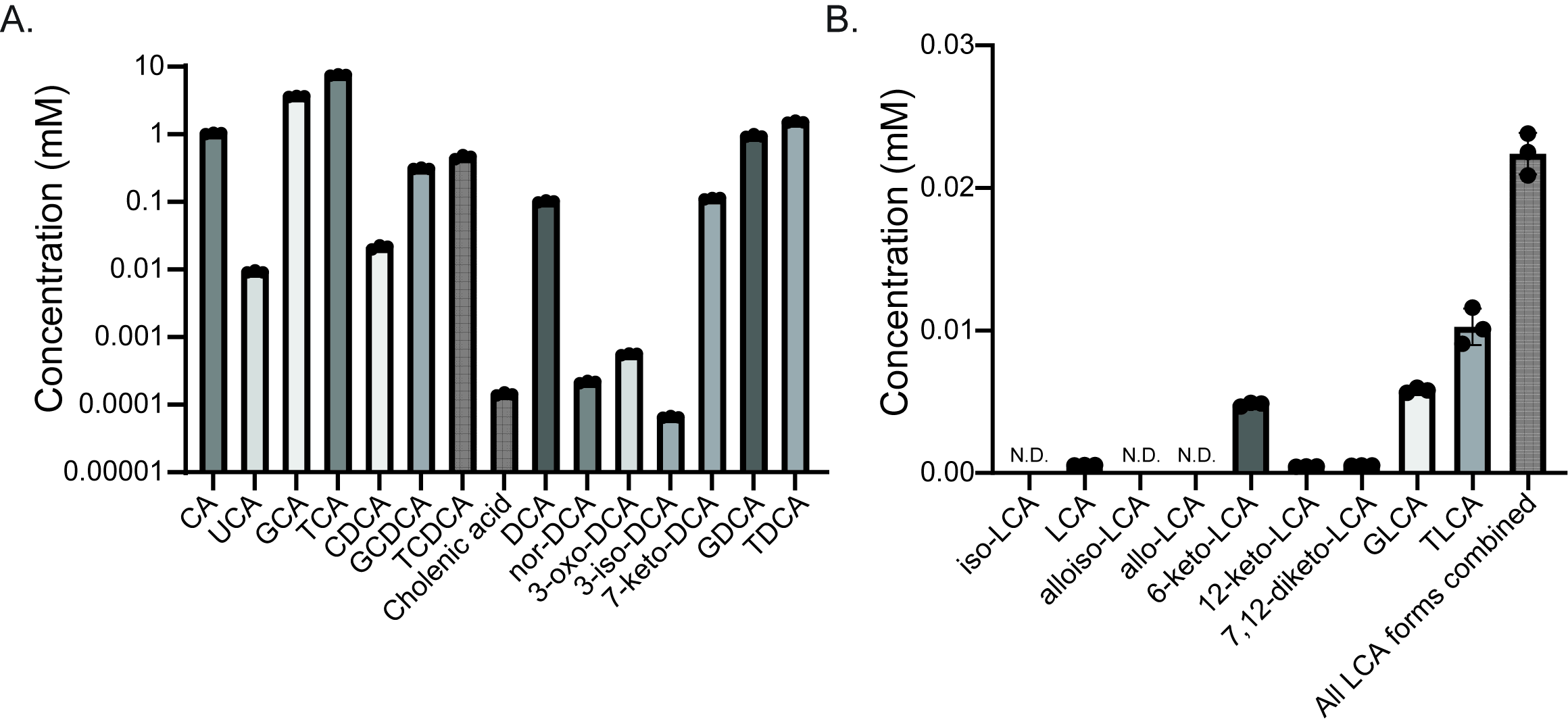

Supplement: Fig. S5 — Compositional analysis of bile. [file spectrum.03419-24-s0005.tif]

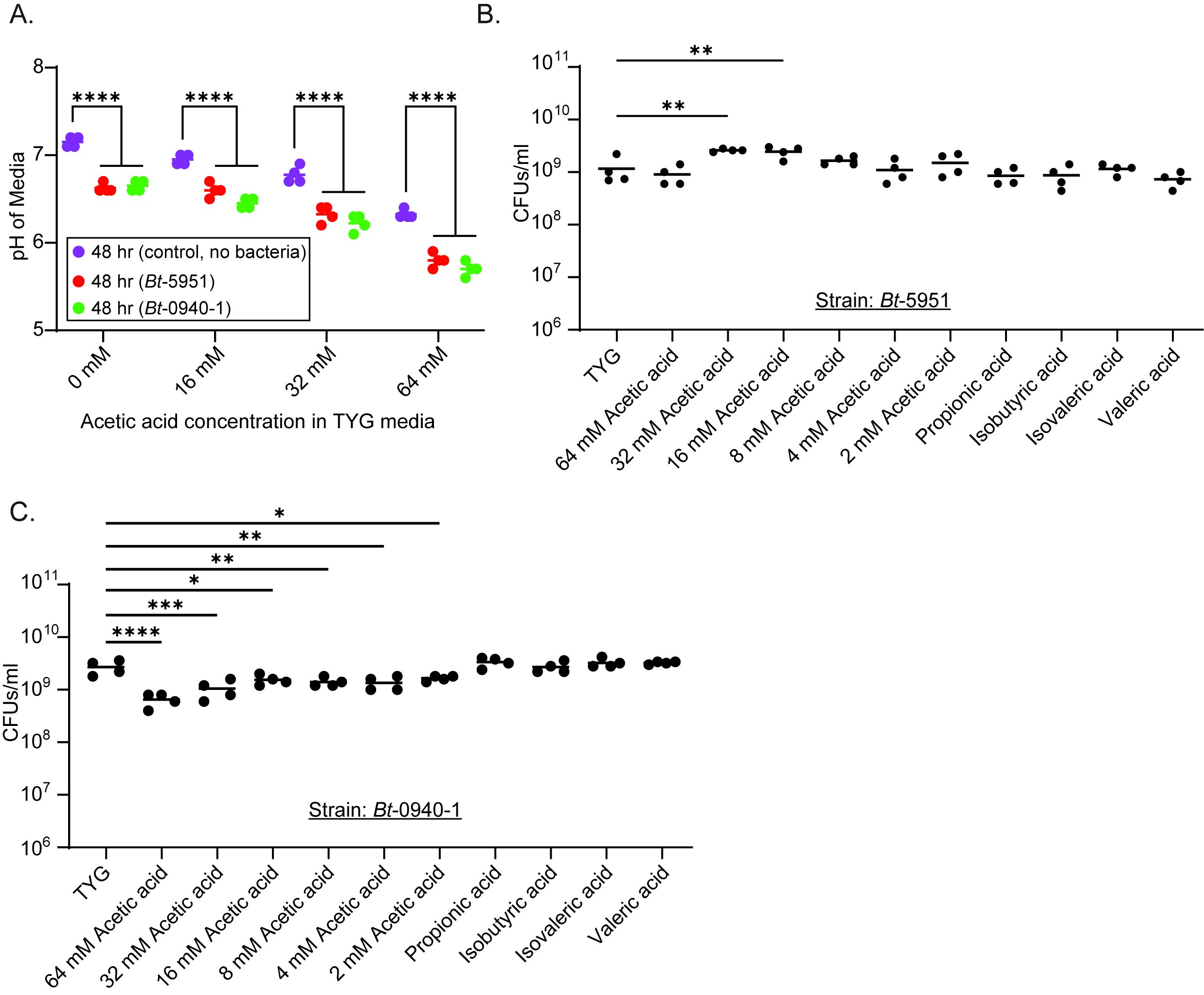

Supplement: Fig. S6 — Short-chain fatty acids do not substantially affect viability. [file spectrum.03419-24-s0006.tif]
