# Supplementary material for: Identification of strain-specific cues that regulate biofilm formation in Bacteroides thetaiotaomicron
Source: Microbiol Spectr. 2025 Aug 18;13(10):e03419-24. doi: 10.1128/spectrum.03419-24 (PMC12502800; doi:10.1128/spectrum.03419-24)
Supplement: Legends — Supplemental figure legends. [file spectrum.03419-24-s0007.pdf]

Figure S1- Replicates of biofilm formation in TYG media, related to Figure 1

(A-C) Independent replicates for the validation of the biofilm assay in TYG media, reflecting the natural variation that occurs in the magnitude of biofilm formation, but reflective of the maintenance of the same pattern between individual strains. All biofilms were quantified at 48 hours post inoculation using a crystal violet assay.

Bars show the mean and each point represents an individual technical replicate. Statistical significance was determined using one-way ANOVA with Dunnet's multiple comparisons test, comparisons made to strain *Bt*-VPI-5482. p values of <0.05 were considered statistically significant, **, $p<0.01$ and ****, $p<0.0001$ for all graphs shown.

Figure S2-Impact of bile on biofilm formation, related to Figure 2

(A,B) Biofilm formation in TYG media with 1% w/v bile after 48 hours of growth for strains *Bt*-VPI-5482 (A) and *Bt*-0940-1 (B). DNase I, RNase A, and Proteinase K enzymes were added to assay biofilm formation induced by bile in the presence of these enzymes. Data is representative of 2 independent experiments.

(C,D) Biofilm formation for strain *Bt*-0940-1 (C) or *Bt*-VPI-5482 (D) grown in TYG for 48 hours with bile acids added at 0.5 mM or bile at 1% w/v. All bile acids induced significantly lower biofilm than TYG, except hyodeoxycholic acid (HDCA) and taurodeoxycholic acid (TDCA). Other bile acids tested include DCA=deoxycholic acid, CA=cholic acid, acid, CDCA=chenodeoxycholic acid, UDCA=ursodeoxycholic acid, GCDCA=glycochenodexocycholic acid, TCDCA=taurochenodexocycholic acid, TCA=taurocholic acid, GCA=glycocholic acid, and LCA= lithocholic acid.

(E) Biofilm formation of strain *Bt*-5951 at 48 hours in TYG media containing the indicated doses of hyodeoxycholic acid (HDCA), ursodeoxycholic acid (UDCA), taurodeoxycholic acid (TDCA), and deoxycholic acid (DCA). Data is representative of 2 independent experiments.

(F) Biofilm assay of lithocholic acid (LCA) added to TYG media without added bacteria at the indicated concentrations. Data is representative of 2 independent experiments.

(G) Biofilm assay of strain *Bt*-5951 grown in TYG or TYG in the presence of 1% (w/v) ethanol (EtOH) or 0.5% DMSO (w/v).

Bars show the mean and each point represents an individual technical replicate. Statistical significance was determined using a one-way ANOVA with Dunnet's multiple comparisons test, comparisons made to 1% w/v bile in (A, B), or compared to TYG (C-E) or without EtOH and DMSO added (G). *p* values of <0.05 were considered statistically significant **, p<0.01 and ***, p<0.001 and ****, p<0.0001.

Figure S3- Bile and lithocholic acid induced biofilm formation across all strains, related to Figure 3

(A) *Bacteroides thetaiotaomicron* strains were grown in 1% w/v bile (pink), 0.5 mM lithocholic acid (LCA, teal), or TYG media (black). Individual points indicate technical replicates. Data are representative of two independent experiments.

Statistical significance was determined using a one-way ANOVA with Dunnet's multiple comparisons test, comparisons made to TYG in (A, B), *p* values of <0.05 were considered statistically significant: *, *p*<0.05, **, *p*<0.01 and ***, *p*<0.001 and ****, *p*<0.0001..

Figure S4- Imaging of bile and lithocholic acid induced biofilm formation, related to Figure 3

(A-C) Confocal imaging of biofilms after 48 hours of growth in 1% w/v bile (A), TYG media (B), or 0.5 mM LCA (C). Images are processed z-stacks. Individual channels and merged are shown (the merged channel is also shown in Figure 3D-F). All images were obtained in the same manner and the scale bar is equal to 50 µm. Orientation is the same in (A-C) with the SYTO-9 (green channel) on the left, propidium iodide (PI, red channel) in the middle, and the merged channel on the right.

Figure S5- Compositional analysis of bile, related to Figure 4

(A,B) Mass spectrometry analysis of 1% w/v commercially available bile used to induce biofilm formation. Data shown are 3 technical replicates showing bile acid analyates detected above a calibrated limit of detection. Both (A) and (B) are from the same sample of bile, but have been separated here for ease of viewing into non-LCA bile acids (A) and LCA bile acids (B) as the concentration of LCA bile acids are orders of magnitude lower. In (B), we are showing a bar at the far right of the graph that has pooled all LCA forms displayed on this panel to show the total sum of all LCA epimers and conjugated forms.

Figure S6-Short-chain fatty acids do not substantially affect viability, related to Figure 5

(A) pH measurements of TYG media containing different doses of the short-chain fatty acid acetic acid at the indicated concentrations (mM) after 48 hours of growth. Data is representative of 2 independent experiments.

(B,C) Colony forming unit (CFU) assay with n=4 biological replicates run concurrently for strains *Bt*-5951 (A) and *Bt*-0940-1 (B). Bacteria were plated on BHI-blood agar after 48 hours of growth in TYG media with the indicated concentration of acetic acid added or propionic acid (8 mM), isobutyric acid (4 mM), isovaleric acid (1 mM), or valeric acid (1 mM). Bars show the mean and each point represents an individual biological replicate.

Statistical significance was determined using a two-way ANOVA with Dunnet's multiple comparisons test, comparisons were made to the no bacteria control at each concentration of acetic acid (A), or to unsupplemented TYG (no acetic acid added) (B,C), ; p values of <0.05 were considered statistically significant, *, $p<0.05$, **, $p<0.01$, ***, $p<0.001$, and ****, $p<0.0001$.
